# Process variables data from the lean vapour compressor campaign at Technology Centre Mongstad

**DOI:** 10.1016/j.dib.2019.104483

**Published:** 2019-09-20

**Authors:** Philip Fosbøl, Randi Neerup, Susana Almeida, Amirali Rezazadeh, Jozsef Gaspar, Anette Knarvik, Nina Flø

**Affiliations:** a Center for Energy Resources Engineering (CERE), Department of Chemical Engineering, Technical University of Denmark (DTU), Søltofts Plads, Building 229, 2800, Kongens Lyngby, Denmark; b Technology Centre Mongstad (TCM DA), 5954, Mongstad, Norway

**Keywords:** Lean vapor compression (LVC), Specific reboiler duty (SRD), Monoethanolamine (MEA), Carbon capture and storage (CCS), Technology Centre Mongstad (TCM), Process optimization

## Abstract

The lean vapor compressor (LVC) unit at Technology Centre Mongstad (TCM), Norway has been tested. The aim of this research has been to create knowledge on the process performance of LVC on the CO_2_ capture efficiency and energy profile of the TCM plant. The data presented in this paper is supplementary to the study “Results of the fourth Technology Centre Mongstad campaign: LVC testing” [1]. The dataset gives unique information on the LVC campaign in which 16 cases have been tested with various campaign process parameters such as LVC pressure, solvent flow, inlet flue gas CO_2_ concentration, and stripper pressure. Absorber and stripper process conditions were recorded during these tests and are presented.

**Table:** Specifications Table

Subject	Renewable Energy, Sustainability and the Environment
Specific subject area	Carbon Capture and Storage
Type of data	Tables
How data were acquired	The data were acquired through the comprehensive SCADA system for the process plant. This include information from flow, temperature, pressure transmitters. Samples were taken in order to determine CO_2_ loading through standardized methods.
Data format	Calculated steady state data based on averaged raw data over 5 minutes intervals
Parameters for data collection	The data were collected under standard operational conditions
Description of data collection	The data were collected under standard operational conditions
Data source location	Technology Centre Mongstad, Mongstad, Norway
Data accessibility	With the article
Related research article	Author's name: Philip Fosbøl, Randi Neerup, Susana Almeida, Amirali Rezazadeh, Jozsef Gaspar, Anette Knarvik, Nina Flø Title: Results of the fourth Technology Centre Mongstad campaign: LVC testing Journal: International Journal of Greenhouse Gas Control DOI: https://doi.org/10.1016/j.ijggc.2019.06.025

**Table:** 

**Value of the Data** • The data describes a comprehensive lean vapour compressor testing campaign at the Technology Centre Mongstad, Norway that serves as benchmark in the field of optimizing the carbon capture and storage (CCS) technology. • There are several stakeholders focused on full-scale carbon capture and storage (CCS) demonstration. Many industries around the world could significantly benefit from full-scale implementations of CCS. • The data can be used for the design of future CCS sites, which rely on reducing the energy consumption on the entire process. • The data are unique as it is the first time lean vapour compression tests at the world's largest test facility for CO_2_ capture are presented.

## 1 Data

The data presented in this article is providing the supplementary information from the lean vapor compression (LVC) campaign at Technology Centre Mongstad, Norway. A detailed description of the LVC campaign is presented in the work by Fosbøl et al. [1]. The LVC campaign was performed in June 2018. The campaign was divided into two main phases a base case and a LVC test phase. Cases from 1A to 1F represent the base cases and the cases ranging from 2A to 2F are the LVC test phase. The process variables from the pilot campaign are shown in Table 1. The standard deviations given in the table are based on averaged raw data given over 5 min intervals. The table gives information such as the inlet conditions to absorber and stripper. It also provides details on temperatures around the main heat exchanger.

**Table 1:** Overview of process variables for the 16 cases, 1A to 2F.

Description	Unit		Case 1A-1	Case 1B	Case 1C	Case 1A-2	Case 1D	Case 1E	Case 1F	Case 2A	Case 2B	Case 2C-1	Case 2C-2	Case 2C-3	Case 2D-1	Case 2D-2	Case 2E	Case 2F
CHP Flue gas flow rate into absorber	Sm^3^/h	mean	34985	34983	34996	34997	34985	34984	34995	34996	34986	34988	34989	34995	35001	34991	34996	34991
		stdev	60	45	50	61	63	60	63	63	60	61	42	53	65	47	60	47
CO_2_ concentration into absorber	vol%, dry	mean	13.5	13.7	13.6	13.5	13.7	13.5	11.0	13.9	13.7	13.7	13.7	13.8	13.9	13.7	13.6	11.2
		stdev	0.04	0.06	0.04	0.05	0.03	0.09	0.02	0.03	0.04	0.03	0.04	0.03	0.02	0.03	0.04	0.04
Flue gas temperature into absorber	°C	mean	30.2	30.1	30.0	30.0	30.1	30.0	30.0	30.1	30	30.1	29.9	30.0	29.9	30	30	30.0
		stdev	0.25	0.04	0.04	0.04	0.05	0.04	0.05	0.04	0.04	0.04	0.04	0.04	0.03	0.06	0.04	0.05
Flue gas inlet pressure	barg	mean	0.017	0.017	0.018	0.017	0.018	0.018	0.018	0.017	0.017	0.018	0.018	0.018	0.018	0.019	0.018	0.018
		stdev	6E-05	2E-04	5E-05	3E-04	2E-04	2E-04	7E-18	36E-04	2E-04	7E-18	1E-04	7E-05	3E-05	2E-04	2E-05	1E-17
Lean solvent temperature	°C	mean	54	54.0	54.0	52.5	54.0	54.0	54.0	50.8	54.00	54.0	54.0	54.0	54.0	54.0	54.0	54.0
		stdev	0.23	0.10	0.02	0.4	0.02	0.07	0.02	0.09	0.17	0.1	0.02	0.04	0.03	0.04	0.02	0.03
Lean solvent density	kg/m^3^	mean	1047	1058	1060	1047	1069	1061	1064	1048	1059	1065	1063	1062	1070	1069	1063	1065
		stdev	0.80	0.70	0.26	0.46	0.79	0.32	0.48	0.10	0.19	0.19	0.42	0.09	0.18	0.14	0.15	0.15
Lean solvent flow rate	t/h	mean	120.1	160.7	200.5	120.5	200.6	200.1	201.3	120.2	165.6	200.9	200.8	200.6	201.7	201.7	200.9	202.1
		stdev	0.2	0.2	0.2	0.1	0.5	0.7	0.2	0.3	0.3	1.2	0.3	0.5	0.3	0.3	0.3	0.4
Flue gas temperature out of absorber	°C	mean	31.4	30.7	30.7	34.0	31.4	32.1	31.6	31.6	31.4	30.8	31.5	31.4	32.3	33.7	30.9	31.6
		stdev	0.12	0.06	0.06	0.36	0.12	0.09	0.03	0.14	0.09	0.1	0.15	0.03	0.15	0.14	0.06	0.04
CO_2_ out of the absorber	vol%, wet	mean	1.5	1.7	1.5	1.6	3.1	1.5	1.2	1.6	1.6	1.9	1.7	1.6	3.1	2.9	1.5	1.3
		stdev	0.06	0.08	0.02	0.09	0.03	0.04	0.02	0.06	0.04	0.03	0.08	0.01	0.02	0.04	0.03	0.02
Rich amine T out from absorber	°C	mean	43.4	47.6	51.3	43	49.8	51.2	51.7	43.2	48.1	51	50.9	51.4	49.6	49.7	51.4	51.8
		stdev	0.14	0.23	0.04	0.48	0.11	0.09	0.03	0.1	0.06	0.09	0.09	0.04	0.04	0.04	0.05	0.05
Rich amine flow going to the HE	t/h	mean	127.3	168.0	208.0	128.0	207.5	207.5	207.5	128.0	173.0	208.0	208.0	208.0	208.0	208.0	208.0	208.0
		stdev	0.5	0.02	0.3	0.05	0.04	0.06	0.04	0.04	0.03	0.04	0.04	0.03	0.04	0.03	0.03	0.02
Rich amine density to HE	kg/m^3^	mean	1107	1100	1093	1104	1098	1094	1090	1107	1100	1097	1096	1095	1100	1099	1096	1092
		stdev	1.1	0.77	0.33	0.92	0.60	0.41	0.42	0.13	0.17	0.21	0.29	0.09	0.18	0.17	0.21	0.25
Rich amine out from HE	°C	mean	109.3	108.9	108.3	110.3	106.6	106.8	108.1	95.1	93.9	93.3	96.1	92.1	91.9	94.8	93.1	93.2
		stdev	0.14	0.08	0.06	0.33	0.09	0.1	0.06	0.07	0.06	0.13	0.05	0.05	0.06	0.05	0.04	0.06
Lean amine to HE	°C	mean	120.6	119.1	118.3	120.4	116.7	116.5	117.8	103.2	101.8	101	104.3	99.5	99.8	103.1	100.8	100.7
		stdev	0.03	0.03	0.02	0.04	0.03	0.05	0.03	0.02	0.05	0.06	0.03	0.03	0.05	0.03	0.02	0.05
Lean amine to sea water cooler	°C	mean	51.9	57.3	60.9	52.5	59.3	60.6	61.2	50.8	55.4	58.3	58.6	58.5	56.9	57.4	58.6	59.0
		stdev	0.13	0.19	0.06	0.37	0.08	0.13	0.05	0.09	0.06	0.13	0.08	0.04	0.06	0.07	0.05	0.05
P in stripper bottom	barg	mean	0.98	0.98	0.98	0.98	0.98	0.84	0.98	0.98	0.98	0.98	0.98	0.98	0.98	0.98	0.84	0.98
		stdev	0.001	0.002	0.001	0.001	0.001	0.002	0.001	0.001	0.001	0.002	0.001	0.001	0.001	0.001	0.001	0.001
Temperature in stripper bottom	°C	mean	120.9	119.4	118.5	120.9	116.9	116.7	118.2	120.8	118.6	117.1	117.5	117.6	115	115.2	115.7	116.7
		stdev	0.03	0.03	0.02	0.02	0.05	0.04	0.03	0.01	0.04	0.05	0.11	0.02	0.05	0.05	0.05	0.05
Top stripper outlet temperature	°C	mean	96.9	97.5	98.7	98.1	96.7	97.2	99.9	88.8	88.7	89.1	91.1	88.5	87.2	89.3	89.2	90.8
		stdev	0.12	0.15	0.05	0.11	0.07	0.11	0.07	0.09	0.05	0.08	0.11	0.04	0.08	0.16	0.06	0.08
Top stripper outlet pressure	barg	mean	0.94	0.95	0.95	0.95	0.94	0.80	0.95	0.94	0.95	0.94	0.95	0.95	0.95	0.95	0.80	0.95
		stdev	0.001	0.002	0.002	0.001	0.001	0.002	0.001	0.002	0.001	0.001	0.001	0.001	0.001	0.001	0.001	0.001
Top stripper outlet flow	kg/h	mean	8623	8655	8971	8842	7474	9511	7543	7357	7084	6979	7431	7159	5935	6404	7728	5997
		stdev	40	77	35	55	26	66	41	36	33	49	64	34	52	32	40	43
CO_2_ Outlet overhead system pressure	barg	mean	0.90	0.90	0.90	0.90	0.90	0.75	0.90	0.90	0.90	0.90	0.90	0.90	0.90	0.90	0.75	0.90
			0.0008	0.0008	0.0004	0.0009	0.0003	0.002	0.0007	0.001	0.0005	0.001	0.0008	0.0007	0.0003	0.0006	0.0009	0.0004
Temperature out of reboiler (HE)	°C	mean	123.3	122.7	122.5	123	119.7	118.4	120.1	122.6	121.8	119.6	119.6	119.8	118.4	118.5	117.5	119.3
		stdev	0.09	0.04	0.03	0.03	0.11	0.05	0.02	0.02	0.03	0.04	0.05	0.02	0.03	0.02	0.02	0.03
Pressure out of reboiler (HE)	barg	mean	0.95	0.96	0.96	0.96	0.96	0.83	0.97	0.95	0.96	0.97	0.98	0.97	0.97	0.97	0.83	0.97
		stdev	0.001	0.002	0.002	0.001	0.002	0.002	0.001	0.001	0.001	0.001	0.001	0.001	0.0004	0.001	0.001	0.001
Temperature into reboiler (HE)	°C	mean	120.8	119.1	118.2	120.6	116.7	116.6	117.9	120.5	118.3	116.8	117.2	117.4	114.7	114.9	115.5	116.5
		stdev	0.05	0.03	0.02	0.04	0.04	0.05	0.04	0.02	0.06	0.05	0.12	0.03	0.04	0.04	0.04	0.05
Pressure into reboiler (HE)	barg	mean	1.1	1.2	1.2	1.2	1.2	1.1	1.2	1.2	1.2	1.2	1.2	1.2	1.2	1.2	1.1	1.2
		stdev	0.001	0.002	0.002	0.001	0.004	0.002	0.001	0.001	0.002	0.002	0.001	0.001	0.001	0.001	0.001	0.001
Temperature of steam into reboiler	°C	mean	160.2	157.2	166.7	157.1	159.1	168.5	162.1	158.8	159.2	159.9	154.5	157.6	156.9	155.6	155.4	158
		stdev	3.3	1.1	0.32	0.31	1.1	0.60	0.46	0.17	1.2	0.15	0.23	0.09	0.10	0.32	0.6	0.8
Pressure of steam into reboiler	barg	mean	2.8	2.8	2.9	2.7	2.3	2.5	2.4	2.3	2.3	2.1	2.3	2.2	1.9	2.0	2.0	2.0
		stdev	0.01	0.01	0.01	0.01	0.01	0.02	0.01	0.01	0.01	0.01	0.01	0.01	0.004	0.01	0.01	0.01
Flow of steam into reboiler	kg/h	mean	12088	12629	13282	12141	11527	12628	11649	9652	9770	9960	11011	10147	9089	9723	10656	8898
		stdev	204	98	40	66	58	53	42	41	47	49	97	50	34	38	65	48
Temperature of steam upstream	°C	mean	156.7	150.6	168.2	153.0	159.3	170.4	163.2	160.8	161.1	161.8	154.5	159.4	159.3	157.6	156.6	160.4
		stdev	7.5	4.2	0.4	1.2	1.7	0.6	0.5	0.2	1.4	0.2	0.7	0.2	0.1	0.4	0.9	0.8
Reboiler outlet temperatue, steam	°C	mean	122.7	120.9	119.8	122.0	117.9	117.3	118.8	122.5	120.5	118.1	118.1	118.5	116	116.1	116.2	117.6
		stdev	0.07	0.04	0.04	0.06	0.11	0.07	0.02	0.01	0.12	0.05	0.08	0.02	0.06	0.04	0.04	0.06
Reboiler outlet pressure, steam	barg	mean	2.8	2.8	2.9	2.7	2.3	2.5	2.4	2.3	2.3	2.1	2.2	2.2	1.9	2.0	2.0	2.0
		stdev	0.009	0.01	0.01	0.01	0.01	0.02	0.007	0.007	0.007	0.008	0.013	0.007	0.004	0.006	0.008	0.008
Condenser bottom flow	kg/h	mean	2878	2977	3254	3123	2484	3415	2893	1746	1689	1708	1999	1706	1413	1619	1991	1600
		stdev	34	39	17	36	16	21	23	20	15	19	31	20	7	13	19	14
Condenser bottom temperature	°C	mean	16.7	16	16.4	16.1	16.4	16.3	15.7	16.7	16.7	16.4	16.4	16.1	16.7	16.3	16.7	15.7
		stdev	0.06	0.13	0.03	0.09	0.17	0.12	0.1	0.07	0.07	0.05	0.06	0.02	0.08	0.37	0.02	0.06
Condenser outlet temperature, hot side	°C	mean	17.5	17.0	17.5	17.0	17.4	17.3	17.5	16.8	17.5	16.9	17.5	16.7	17.6	17.3	17.5	16.5
		stdev	0.13	0.14	0.06	0.13	0.2	0.18	0.11	0.11	0.1	0.13	0.12	0.08	0.08	0.3	0.09	0.09
Flow of seawater to condenser inlet	kg/h	mean	92278	98716	104193	105040	81885	128612	90363	55781	47816	49786	54966	51890	34865	40070	59047	43988
		stdev	852	117	617	106	508	610	554	57	484	49	731	41	391	487	450	544
Temperature of seawater to condenser inlet	°C	mean	7.7	7.6	7.6	7.7	8.6	8.4	8.4	7.7	7.7	7.7	7.7	7.6	7.7	7.7	7.8	8.0
		stdev	0.02	0.006	0.02	0.01	0.17	0.11	0.02	0.008	0.03	0.05	0.01	0.02	0.01	0.02	0.07	0.06
Temperature of seawater to condenser outlet	°C	mean	28.3	27.4	28.0	27.2	28.4	25.7	29	28.7	31.0	30.1	30.3	29.1	33.6	32.5	29.6	31.2
		stdev	0.22	0.21	0.07	0.18	0.22	0.21	0.17	0.19	0.18	0.18	0.23	0.13	0.17	0.67	0.18	0.23
Flow of CO_2_ out of condenser drum	kg/h	mean	7553	7468	7404	7443	6589	7408	6038	7726	7473	7290	7419	7539	6627	6627	7409	6114
		stdev	21	33	26	47	23	48	27	33	29	47	49	35	26	23	33	45
Amine flash vessel pressure	barg	mean	1.0	1.0	1.0	1.0	1.0	0.87	1.0	0.046	0.046	0.047	0.20	−0.0061	0.043	0.19	0.046	0.044
		stdev	0.001	0.001	0.001	0.003	0.002	0.001	0.001	0.001	0.002	0.002	0.001	0.001	0.001	0.001	0.001	0.002
Flash vessel inlet temperature	°C	mean	112.0	110.3	108.5	111	107	108.8	108	101.7	100.8	100.3	103.8	99	99.1	102.6	100.3	100.1
		stdev	0.07	0.09	0.06	0.68	0.12	0.06	0.16	0.03	0.05	0.06	0.03	0.03	0.05	0.03	0.03	0.05
Lean amine temperature to antisurge HE	°C	mean	120.8	119.2	118.4	120.5	116.8	116.6	117.9	102.3	101.4	100.8	104.1	99.4	99.5	102.9	100.7	100.5
		stdev	0.05	0.03	0.03	0.03	0.03	0.05	0.04	0.02	0.04	0.05	0.04	0.02	0.05	0.03	0.02	0.05
Lean amine flow to flash vessel	t/h	mean	114.9	152.6	190.3	116.0	188.5	189.6	190.6	117.7	160.4	193.4	193.2	194.6	192.6	191.7	193.5	194.6
		stdev	0.5	0.3	0.3	0.6	1.0	0.4	0.3	0.4	0.5	0.8	0.7	0.5	0.4	0.7	0.8	0.8
Inlet pressure to compressor	barg	mean	0.95	0.95	0.94	0.95	0.94	0.80	0.94	−0.066	−0.069	−0.060	0.091	−0.13	−0.077	0.089	−0.061	−0.072
		stdev	0.005	0.0006	0.0009	0.004	0.001	0.002	0.001	0.001	0.002	0.002	0.001	0.001	0.001	0.002	0.001	0.001
Inlet temperature to compressor	°C	mean	35.6	45.4	44.5	42.7	60.0	42.3	62.5	100.4	99.5	98.9	102.8	97.3	97.6	101.6	99.0	98.6
		stdev	0.74	0.52	0.60	10.2	0.25	5.8	0.73	0.02	0.04	0.05	0.03	0.02	0.05	0.04	0.03	0.05
Outlet pressure from compressor	barg	mean	0.92	0.87	0.86	0.88	0.88	0.70	0.87	1.04	1.05	1.06	1.00	1.04	0.99	0.97	0.87	1.01
		stdev	0.01	0.009	0.005	0.005	0.004	0.004	0.002	0.003	0.009	0.003	0.001	0.002	0.002	0.004	0.002	0.006
Outlet temperature from compressor	°C	mean	25.0	20.9	17.8	18.9	29.3	22.0	41.3	192.2	190.7	188.9	171.9	197.5	188.8	170.7	178.3	189.4
		stdev	0.86	0.17	0.16	1.0	0.33	1.0	3.0	0.06	0.15	0.28	0.18	0.07	0.06	0.2	0.09	0.43
Lean loading^a^	mol/mol		0.215	0.265	0.290	-b	0.318	0.273	0.292	0.201	0.266	0.284	0.280	0.285	0.318	0.318	0.288	0.301
Rich loading^a^	mol/mol		0.483	0.524	0.507	-b	0.507	0.486	0.467	0.543	0.513	0.493	0.488	0.483	0.493	0.501	0.496	0.467
CO_2_ capture	%	mean	90.1	88.9	89.7	88.9	78.7	89.8	89.5	89.8	89.4	87.4	88.4	89.5	79.2	80.5	89.6	89.4
		stdev	0.4	0.5	0.1	0.6	0.2	0.3	0.2	0.2	0.3	0.2	0.5	0.1	0.1	0.02	0.2	0.2
Q^c^_SRD_	MJ/kg CO_2_	mean	3.60	3.77	4.00	3.66	3.90	3.83	4.34	2.79	2.90	3.04	3.29	3.00	3.03	3.23	3.20	3.26
		stdev	0.06	0.04	0.02	0.04	0.03	0.04	0.02	0.02	0.02	0.02	0.02	0.02	0.02	0.02	0.03	0.02
Q^d^_LVC_	GJ electric/ton CO_2_	mean	0.000	0.000	0.000	0.000	0.000	0.000	0.000	0.19	0.19	0.19	0.14	0.21	0.21	0.14	0.15	0.24
		stdev	0.000	0.000	0.000	0.000	0.000	0.000	0.000	0.008	0.001	0.001	0.001	0.001	0.001	0.002	0.001	0.001
Antisurge cooler inlet cold side temp	°C	mean	121	119	118	121	117	117	118	102	101	101	104	99	99	103	101	101
		stdev	0.05	0.03	0.03	0.03	0.03	0.05	0.04	0.02	0.04	0.05	0.04	0.02	0.05	0.03	0.02	0.05
Antisurge cooler outlet cold side temp	°C	mean	120.6	119.1	118.3	120.4	116.7	116.5	117.8	103.2	101.8	101	104.3	99.5	99.8	103.1	100.8	100.7
		stdev	0.03	0.03	0.02	0.04	0.03	0.05	0.03	0.02	0.05	0.06	0.03	0.03	0.05	0.03	0.02	0.05
Antisurge cooler outlet cold side flow	t/h	mean	117.6	161.7	201.52	122.7	199.4	200.6	201.4	120.8	164.3	198.1	198.9	198.1	197.8	198.3	198.5	199.1
		stdev	0.26	0.26	0.27	0.13	0.69	0.76	0.36	0.38	0.35	0.11	0.33	0.56	0.3	0.32	0.32	0.36
Seawater flow to antisurge	kg/h	mean	33294	33118	33329	33189	33173	32818	33242	33243	33308	33253	33362	33301	33350	33399	33331	33468
		stdev	72	32	418	251	173	158	161	167	64	191	152	201	155	156	212	154
Temp. of seawater out of antisurge	°C	mean	7.8	7.7	7.6	7.7	8.6	8.4	8.4	8.9	8.7	8.8	8.8	8.4	8.3	8.6	8.7	9.0
		stdev	0.09	0.03	0.03	0.05	0.15	0.09	0.02	0.01	0.05	0.05	0.02	0.01	0.02	0.02	0.07	0.04
Temp. of seawater to antisurge inlet	°C	mean	7.7	7.6	7.6	7.7	8.6	8.4	8.4	7.7	7.7	7.7	7.7	7.6	7.7	7.7	7.8	8.0
		stdev	0.02	0.006	0.02	0.01	0.17	0.11	0.02	0.008	0.03	0.05	0.01	0.02	0.01	0.02	0.07	0.06

a Uncertainty on the lean- and rich loading determination of 4%.

b Lean and rich loading not measured for case 1A-2.

c SRD is thermal energy consumption.

d LVC is electrical energy consumption.

The absorber temperature profiles for the base cases are given in Table 2, Table 3. The stripper temperature profiles for the base and the LVC cases are listed in Table 4, Table 5 respectively.

**Table 2:** Absorber temperature profiles for base cases.

h (m)	Position of temperature probe^a^	Case 1A-1		Case 1A-2		Case 1B		Case 1C		Case 1D		Case 1E		Case 1F	
		T (°C)	Std. Dev. (°C)	T (°C)	Std. Dev. (°C)	T (°C)	Std. Dev. (°C)	T (°C)	Std. Dev. (°C)	T (°C)	Std. Dev. (°C)	T (°C)	Std. Dev. (°C)	T (°C)	Std. Dev. (°C)
0	Below lower packing	48	0.12	48	0.37	54	0.36	58	0.3	56	0.2	59	0.22	59	0.27
0.5	a	48	0.14	47	0.38	53	0.34	57	0.18	55	0.18	57	0.23	57	0.21
	b	39	0.14	39	0.29	45	0.09	48	0.05	47	0.06	47	0.11	49	0.06
	c	36	0.19	36	0.15	42	0.09	46	0.06	45	0.05	46	0.12	47	0.07
	d	36	0.18	36	0.1	40	0.07	44	0.09	44	0.1	44	0.1	45	0.07
1.5	a	48	0.13	48	0.41	54	0.17	58	0.1	56	0.12	57	0.13	58	0.11
	b	38	0.13	39	0.27	45	0.08	49	0.05	48	0.06	48	0.11	50	0.04
	c	47	0.11	47	0.24	57	0.22	64	0.08	60	0.11	64	0.19	66	0.05
	d	38	0.13	38	0.14	45	0.1	50	0.08	49	0.07	50	0.14	51	0.05
2.5	a	57	0.18	56	0.53	64	0.26	67	0.09	64	0.15	66	0.16	67	0.08
	b	41	0.1	41	0.34	49	0.1	53	0.06	52	0.07	53	0.12	54	0.05
	c	48	0.11	48	0.47	55	0.15	58	0.08	57	0.13	58	0.11	59	0.06
	d	46	0.1	46	0.36	55	0.13	59	0.1	57	0.1	59	0.15	61	0.04
3.5	a	55	0.17	55	0.55	62	0.22	66	0.08	63	0.14	65	0.13	66	0.06
	b	46	0.1	46	0.45	54	0.13	58	0.07	56	0.07	58	0.14	59	0.05
	c	43	0.09	43	0.26	52	0.14	57	0.07	56	0.07	57	0.17	59	0.04
	d	46	0.1	46	0.23	55	0.18	62	0.1	59	0.09	61	0.22	63	0.05
4.5	a	62	0.24	61	0.61	68	0.27	71	0.08	68	0.13	71	0.14	71	0.05
	b	52	0.15	52	0.56	61	0.2	64	0.08	62	0.1	64	0.16	65	0.06
	c	44	0.09	44	0.2	53	0.14	60	0.11	57	0.09	59	0.2	61	0.05
	d	48	0.11	48	0.34	59	0.22	66	0.08	62	0.08	65	0.2	67	0.07
5.5	a	63	0.27	62	0.63	69	0.29	72	0.08	69	0.13	72	0.14	72	0.05
	b	52	0.16	51	0.55	61	0.2	65	0.08	62	0.09	65	0.16	66	0.08
	c	49	0.13	49	0.25	59	0.21	66	0.09	63	0.12	65	0.22	67	0.08
	d	47	0.1	46	0.23	56	0.19	63	0.1	60	0.09	63	0.22	65	0.09
6.5	a	64	0.28	63	0.64	70	0.28	73	0.06	70	0.13	73	0.13	73	0.06
	b	55	0.18	54	0.62	64	0.23	68	0.07	65	0.1	68	0.17	69	0.06
	c	49	0.12	48	0.25	58	0.44	65	0.1	62	0.13	65	0.34	67	0.08
	d	51	0.13	51	0.37	63	0.25	69	0.08	65	0.09	68	0.22	69	0.04
7.5	a	68.8	0.28	67.3	0.63	73.8	0.26	76	0.06	72.5	0.12	75.8	0.12	74.8	0.03
	b	57.3	0.22	56.7	0.66	66.8	0.22	70.5	0.08	66.8	0.12	70	0.15	70.6	0.05
	c	55	0.18	54.4	0.56	66.1	0.28	70.6	0.07	66.9	0.1	70.3	0.19	70.7	0.03
	d	54.7	0.16	54	0.37	65.1	0.26	71	0.09	67.1	0.11	70.5	0.18	71.2	0.04
8.5	a	70.4	0.31	68.8	0.66	74.9	0.25	76.8	0.05	73.5	0.11	76.6	0.11	75.3	0.03
	b	64.2	0.3	63	0.73	71.4	0.24	74.1	0.06	70.4	0.12	73.7	0.14	73.3	0.04
	c	57.1	0.21	56.3	0.57	68	0.25	72.4	0.06	68.6	0.11	72	0.16	72	0.04
	d	57.1	0.21	56.6	0.38	67.4	0.25	72.7	0.09	68.9	0.1	72.5	0.15	72.4	0.04
9.5	a	70.5	0.28	69	0.65	74.9	0.22	76.7	0.05	73.4	0.11	76.5	0.11	75	0.03
	b	62.1	0.26	N/A	N/A	70.6	0.22	N/A	N/A	N/A	N/A	N/A	N/A	N/A	N/A
	c	61.3	0.28	60	0.4	70.1	0.29	75.1	0.06	71.2	0.1	74.8	0.15	73.9	0.03
	d	61.7	0.23	60.6	0.54	71.1	0.22	74.8	0.05	71.3	0.11	74.6	0.13	73.7	0.03
10.5	a	73.1	0.26	71.7	0.58	76.5	0.18	77.8	0.04	75	0.09	77.7	0.09	75.8	0.03
	b	67.5	0.28	66.2	0.69	73.7	0.19	75.8	0.04	72.8	0.09	75.7	0.11	74.3	0.02
	c	68.3	0.26	67	0.71	74.2	0.17	76.1	0.04	73.2	0.09	75.9	0.09	74.4	0.02
	d	65.1	0.26	63.9	0.45	72.6	0.24	76.3	0.05	72.9	0.08	76.1	0.12	74.7	0.03
11.5	a	73.5	0.26	72.1	0.49	76.3	0.17	77.8	0.03	75.2	0.08	77.7	0.09	75.6	0.03
	b	72.1	0.24	70.9	0.53	75.7	0.15	77.2	0.03	74.8	0.07	77.2	0.09	75.3	0.02
	c	71	0.22	69.7	0.52	75	0.14	76.9	0.03	74.4	0.07	76.8	0.08	74.8	0.03
	d	67.7	0.27	66.3	0.46	73.9	0.19	76.6	0.04	73.8	0.07	76.5	0.09	74.8	0.02
12	Below middle packing	69	0.29	67.8	0.44	74.7	0.23	77.3	0.04	74.1	0.09	77.2	0.09	75.3	0.02
12.5	a	73.7	0.19	72.6	0.38	76.4	0.1	77.7	0.03	75.5	0.06	77.5	0.08	75.2	0.02
	b	72.6	0.2	71.5	0.43	75.8	0.12	77.4	0.03	75.2	0.06	77.4	0.09	75.2	0.02
	c	69.8	0.27	68.5	0.47	74.6	0.2	77.4	0.04	74.6	0.08	77.3	0.09	75.3	0.03
	d	71.9	0.21	70.7	0.42	75.3	0.14	77.5	0.03	75.2	0.06	77.5	0.09	75.3	0.02
13.5	a	75.2	0.13	74.4	0.29	77.2	0.07	78.1	0.03	76.5	0.05	78.1	0.08	75.6	0.02
	b	74.3	0.15	73.4	0.34	76.6	0.08	77.7	0.03	75.9	0.05	77.7	0.08	75.4	0.02
	c	71.6	0.27	70.3	0.46	75.5	0.16	77.7	0.03	75.2	0.07	77.6	0.08	75.5	0.02
	d	73.8	0.17	72.8	0.34	76.4	0.1	78	0.03	76	0.05	78	0.08	75.7	0.02
14.5	a	75.9	0.12	75.2	0.23	77.5	0.05	78.2	0.03	76.8	0.05	78.2	0.08	75.7	0.02
	b	75.3	0.14	74.6	0.26	77.2	0.06	78.1	0.03	76.6	0.04	78.2	0.08	75.7	0.02
	c	73	0.23	71.9	0.38	76	0.13	77.7	0.03	75.6	0.07	77.6	0.08	75.4	0.02
	d	74.7	0.16	73.8	0.28	76.8	0.08	78.1	0.03	76.4	0.04	78.1	0.08	75.7	0.02
15.5	a	76.7	0.06	76.2	0.14	77.8	0.04	78.1	0.03	77.1	0.03	78.1	0.08	75.2	0.03
	b	76.1	0.08	75.6	0.18	77.5	0.04	78.2	0.03	77	0.04	78.2	0.08	75.7	0.02
	c	69.3	2.4	73.4	0.3	76.7	0.09	78	0.03	76.6	0.38	77.9	0.08	75.6	0.02
	d	75.6	0.1	74.9	0.19	77	0.05	78	0.03	76.7	0.03	78	0.08	75.5	0.02
16.5	a	77	0.06	76.6	0.12	78	0.04	78	0.04	77	0.04	78	0.09	74.5	0.05
	b	76.7	0.06	76.3	0.12	77.8	0.03	78.1	0.03	77.2	0.03	78.2	0.08	75.4	0.02
	c	75.5	0.11	74.8	0.2	77.1	0.05	78	0.03	76.6	0.04	78	0.08	75.3	0.02
	d	76.6	0.07	76.1	0.13	77.8	0.04	78.4	0.03	77.4	0.03	78.4	0.09	75.6	0.03
17.5	a	73	0.19	72.2	0.41	70.5	0.29	67.1	0.28	66.5	0.37	67.4	0.41	62.3	0.13
	b	75	0.12	74	0.22	73.3	0.12	70.8	0.16	70.3	0.12	71.3	0.18	66.7	0.16
	c	73.1	0.12	72.3	0.18	72.7	0.19	70.4	0.18	69	0.12	70.4	0.17	66.1	0.08
	d	73.6	0.11	72.8	0.17	72.3	0.1	69.8	0.12	68.9	0.15	70	0.21	65.4	0.12
18	Below upper packing	72.2	0.08	71.2	0.23	71.9	0.04	70.3	0.03	68.9	0.03	70.1	0.08	66.6	0.02

a There are four parallel temperature sensors, where the legends A, B, C, and D refer to the temperature sensor close to the column wall and inside the packing at horizontal 1 m distance from each other. N/A: Data not available

**Table 3:** Absorber temperature profiles for LVC cases.

h (m)	Position of temperature probe^a^	Case 2A		Case 2B		Case 2C-1		Case 2C-2		Case 2C-3		Case 2D-1		Case 2D-2		Case 2E		Case 2F	
		T (°C)	Std. Dev. (°C)	T (°C)	Std. Dev. (°C)	T (°C)	Std. Dev. (°C)	T (°C)	Std. Dev. (°C)	T (°C)	Std. Dev. (°C)	T (°C)	Std. Dev. (°C)	T (°C)	Std. Dev. (°C)	T (°C)	Std. Dev. (°C)	T (°C)	Std. Dev. (°C)
0	Below lower packing	48	0.13	55	0.22	57	0.24	57	0.23	58	0.12	56	0.35	56	0.17	58	0.22	59	0.29
0.5	a	48	0.13	54	0.15	56	0.22	57	0.2	57	0.15	55	0.14	55	0.15	57	0.31	58	0.19
	b	39	0.07	45	0.08	48	0.11	48	0.07	48	0.06	47	0.05	47	0.06	48	0.06	49	0.07
	c	36	0.05	43	0.07	46	0.14	46	0.08	46	0.06	45	0.06	45	0.07	46	0.06	47	0.1
	d	36	0.04	41	0.08	44	0.15	44	0.09	44	0.08	43	0.07	43	0.11	44	0.09	45	0.1
1.5	a	48	0.09	55	0.11	57	0.12	57	0.14	58	0.09	56	0.13	56	0.12	58	0.1	59	0.1
	b	38	0.07	46	0.08	49	0.12	49	0.06	49	0.05	48	0.06	48	0.07	49	0.06	50	0.06
	c	47	0.06	58	0.15	63	0.2	63	0.25	64	0.08	61	0.07	61	0.05	64	0.08	66	0.12
	d	38	0.04	46	0.07	50	0.18	50	0.14	50	0.09	49	0.08	49	0.08	50	0.06	51	0.1
2.5	a	57	0.15	64	0.14	66	0.12	66	0.22	67	0.06	64	0.11	64	0.1	67	0.13	67	0.1
	b	41	0.08	50	0.08	53	0.13	53	0.09	53	0.05	52	0.06	52	0.06	53	0.07	55	0.06
	c	48	0.09	56	0.12	58	0.13	58	0.09	59	0.06	57	0.04	57	0.06	58	0.07	60	0.08
	d	46	0.07	55	0.13	59	0.13	59	0.23	59	0.07	57	0.11	57	0.09	59	0.1	61	0.1
3.5	a	55	0.12	63	0.12	65	0.13	65	0.18	66	0.06	63	0.08	63	0.07	66	0.08	67	0.09
	b	45	0.1	55	0.1	58	0.14	58	0.13	58	0.06	56	0.04	56	0.06	58	0.08	60	0.06
	c	42	0.06	53	0.08	57	0.18	57	0.13	57	0.07	56	0.06	56	0.07	57	0.07	59	0.09
	d	45	0.06	56	0.11	61	0.19	61	0.23	62	0.1	59	0.1	59	0.06	62	0.1	64	0.09
4.5	a	62	0.2	69	0.15	70	0.12	70	0.23	71	0.05	67	0.09	68	0.07	71	0.09	71	0.07
	b	52	0.13	61	0.14	64	0.13	64	0.2	64	0.06	62	0.04	62	0.05	64	0.08	66	0.08
	c	44	0.04	54	0.08	59	0.21	59	0.2	60	0.09	57	0.07	57	0.09	60	0.08	61	0.1
	d	48	0.09	61	0.14	65	0.19	65	0.21	66	0.08	63	0.09	63	0.07	66	0.12	67	0.08
5.5	a	63	0.21	70	0.16	72	0.11	72	0.21	72	0.05	69	0.07	69	0.05	72	0.09	72	0.06
	b	51	0.13	62	0.14	64	0.14	64	0.21	65	0.06	62	0.04	62	0.06	65	0.08	66	0.07
	c	49	0.07	60	0.15	65	0.2	65	0.26	66	0.09	63	0.13	63	0.09	66	0.12	67	0.12
	d	46	0.08	58	0.11	63	0.22	63	0.26	63	0.09	60	0.06	61	0.08	63	0.07	65	0.1
6.5	a	64	0.21	71	0.16	73	0.11	73	0.2	73	0.05	69	0.06	70	0.06	73	0.07	73	0.05
	b	55	0.15	65	0.17	67	0.14	67	0.24	68	0.06	65	0.04	65	0.06	68	0.08	69	0.06
	c	48	0.06	59	0.13	65	0.24	64	0.38	66	0.13	62	0.07	63	0.09	66	0.12	67	0.11
	d	51	0.12	64	0.15	68	0.18	68	0.24	69	0.07	65	0.08	65	0.08	69	0.1	69	0.06
7.5	a	68	0.26	74	0.15	75	0.08	75	0.18	76	0.04	72	0.06	73	0.06	76	0.07	75	0.04
	b	57	0.17	67	0.17	70	0.13	70	0.24	71	0.06	67	0.04	67	0.06	70	0.08	71	0.06
	c	55	0.17	67	0.16	70	0.16	70	0.22	71	0.07	67	0.07	67	0.07	71	0.1	71	0.06
	d	54	0.1	66	0.14	70	0.16	70	0.26	71	0.08	67	0.09	67	0.06	71	0.1	71	0.08
8.5	a	70	0.24	75	0.14	76	0.07	76	0.16	77	0.03	73	0.06	74	0.05	77	0.06	75	0.03
	b	64	0.22	72	0.16	73	0.1	73	0.21	74	0.04	70	0.04	71	0.06	74	0.06	74	0.05
	c	57	0.17	69	0.15	72	0.14	72	0.21	72	0.06	69	0.07	69	0.07	72	0.09	72	0.05
	d	57	0.12	68	0.17	72	0.15	72	0.22	73	0.06	69	0.1	69	0.06	73	0.07	73	0.06
9.5	a	70	0.25	75	0.12	76	0.07	76	0.14	77	0.04	73	0.06	74	0.05	77	0.05	75	0.03
	b	N/A	N/A	N/A	N/A	N/A	N/A	N/A	N/A	N/A	N/A	N/A	N/A	N/A	N/A	N/A	N/A	N/A	N/A
	c	61	0.15	71	0.17	74	0.12	74	0.2	75	0.06	71	0.07	71	0.05	75	0.06	74	0.04
	d	61	0.19	72	0.14	74	0.11	74	0.17	75	0.05	71	0.06	72	0.07	75	0.07	74	0.04
10.5	a	73	0.23	77	0.1	77	0.06	77	0.11	78	0.03	75	0.05	75	0.05	78	0.03	76	0.03
	b	67	0.22	74	0.13	75	0.07	75	0.13	76	0.04	73	0.07	73	0.05	76	0.05	75	0.03
	c	68	0.22	75	0.12	76	0.07	76	0.12	76	0.04	73	0.06	73	0.04	76	0.05	75	0.03
	d	64	0.17	73	0.15	76	0.09	76	0.17	76	0.05	73	0.08	73	0.05	76	0.05	75	0.04
11.5	a	73	0.21	77	0.09	77	0.05	77	0.09	78	0.03	75	0.05	75	0.04	78	0.03	76	0.04
	b	72	0.19	76	0.09	77	0.05	77	0.1	77	0.03	75	0.06	75	0.04	77	0.03	75	0.03
	c	71	0.17	75	0.09	77	0.05	76	0.09	77	0.03	74	0.05	74	0.04	77	0.03	75	0.03
	d	67	0.19	74	0.11	76	0.07	76	0.12	77	0.04	74	0.07	74	0.05	77	0.04	75	0.03
12	Below middle packing	68	0.24	75	0.14	77	0.05	77	0.12	77	0.03	74	0.05	74	0.05	77	0.04	76	0.03
12.5	a	74	0.16	77	0.06	78	0.04	77	0.07	78	0.03	76	0.05	76	0.04	78	0.03	75	0.03
	b	72	0.17	76	0.07	77	0.04	77	0.07	78	0.03	75	0.05	75	0.04	77	0.03	75	0.03
	c	69	0.22	75	0.11	77	0.06	77	0.1	78	0.03	74	0.05	75	0.05	77	0.04	76	0.03
	d	71	0.19	76	0.08	77	0.05	77	0.07	78	0.03	75	0.04	75	0.04	77	0.03	75	0.03
13.5	a	75	0.11	77	0.04	78	0.03	78	0.04	78	0.03	76	0.05	76	0.04	78	0.03	76	0.04
	b	74	0.13	77	0.05	78	0.04	77	0.06	78	0.03	76	0.05	76	0.04	78	0.03	76	0.04
	c	71	0.22	76	0.09	77	0.05	77	0.08	78	0.03	75	0.05	75	0.04	78	0.03	76	0.03
	d	74	0.16	77	0.06	78	0.04	78	0.03	78	0.03	76	0.05	76	0.04	78	0.03	76	0.03
14.5	a	76	0.09	78	0.04	78	0.03	78	0.04	78	0.03	77	0.05	77	0.04	78	0.03	76	0.04
	b	75	0.1	77	0.04	78	0.03	78	0.04	78	0.03	76	0.05	76	0.03	78	0.03	76	0.04
	c	73	0.17	76	0.07	77	0.04	77	0.07	78	0.03	75	0.05	75	0.04	78	0.03	76	0.04
	d	74	0.13	77	0.05	78	0.03	78	0.04	78	0.03	76	0.05	76	0.03	78	0.03	76	0.04
15.5	a	77	0.05	78	0.03	78	0.03	78	0.02	78	0.03	77	0.05	77	0.04	78	0.04	75	0.05
	b	76	0.06	78	0.03	78	0.03	78	0.03	78	0.03	77	0.05	77	0.04	78	0.04	76	0.04
	c	74	0.13	77	0.05	78	0.03	78	0.04	78	0.03	76	0.05	76	0.04	78	0.03	76	0.04
	d	76	0.08	77	0.03	78	0.03	78	0.04	78	0.03	76	0.06	76	0.03	78	0.03	76	0.04
16.5	a	77	0.04	78	0.03	78	0.04	78	0.03	78	0.04	77	0.04	77	0.03	78	0.04	75	0.07
	b	77	0.04	78	0.03	78	0.03	78	0.03	78	0.03	77	0.04	77	0.04	78	0.04	76	0.04
	c	76	0.08	77	0.04	78	0.03	78	0.03	78	0.03	76	0.05	76	0.03	78	0.04	75	0.05
	d	77	0.05	78	0.03	78	0.03	78	0.03	79	0.03	77	0.05	77	0.03	78	0.04	76	0.05
17.5	a	72	0.15	70	0.41	67	0.34	66	0.37	67	0.25	66	0.23	66	0.23	67	0.28	63	0.18
	b	75	0.08	73	0.14	71	0.15	71	0.13	71	0.15	70	0.06	70	0.11	71	0.14	67	0.16
	c	73	0.11	72	0.26	70	0.16	71	0.1	71	0.13	69	0.09	69	0.08	70	0.14	66	0.13
	d	73	0.07	72	0.13	70	0.18	70	0.12	70	0.15	69	0.11	69	0.14	70	0.15	65	0.14
18	Below middle packing	71	0.09	72	0.06	70	0.04	70	0.03	70	0.03	69	0.04	69	0.05	70	0.04	67	0.06

a There are four parallel temperature sensors, where the legends A, B, C, and D refer to the temperature sensor close to the column wall and inside the packing at horizontal 1 m distance from each other. N/A: Data not available

**Table 4:** Stripper temperature profiles for base cases.

h (m)	Position of temperature probe^a^	Case 1A-1		Case 1A-2		Case 1B		Case 1C		Case 1D		Case 1E		Case 1F	
		T (°C)	Std. Dev. (°C)	T (°C)	Std. Dev. (°C)	T (°C)	Std. Dev. (°C)	T (°C)	Std. Dev. (°C)	T (°C)	Std. Dev. (°C)	T (°C)	Std. Dev. (°C)	T (°C)	Std. Dev. (°C)
0		121	0.03	121	0.02	119	0.03	118	0.02	117	0.05	117	0.04	118	0.03
0.5	a	120	0.35	118	0.08	117	0.41	115	0.11	112	2.02	114	0.37	110	0.13
	b	120	0.05	120	0.03	119	0.1	118	0.06	114	0.39	115	0.1	114	0.11
	c	119	0.04	118	0.07	113	0.32	113	0.17	103	0.1	107	0.37	111	0.18
	d	N/A	N/A	N/A	N/A	N/A	N/A	N/A	N/A	N/A	N/A	N/A	N/A	N/A	N/A
1.5	a	118	0.06	117	0.15	114	0.71	110	0.19	105	0.2	111	0.6	107	0.1
	b	119	0.06	119	0.03	118	0.21	115	0.12	111	0.63	114	0.13	110	0.17
	c	117	0.08	117	0.09	107	0.41	108	0.23	102	0.03	103	0.21	107	0.16
	d	117	0.11	119	0.07	110	0.24	109	0.17	102	0.04	107	0.27	106	0.11
2.5	a	118	0.05	118	0.1	117	0.53	112	0.26	107	0.27	113	0.14	107	0.17
	b	118	0.07	119	0.05	111	0.29	109	0.18	102	0.04	107	0.28	106	0.09
	c	N/A	N/A	N/A	N/A	N/A	N/A	N/A	N/A	N/A	N/A	N/A	N/A	N/A	N/A
	d	77	57.01	118	0.14	106	0.21	108	0.1	105	0.03	105	0.11	108	0.07
3.5	a	116	0.12	115	0.23	113	1.11	106	0.2	103	0.1	110	0.29	105	0.07
	b	114	0.17	117	0.1	104	0.23	105	0.07	102	0.03	103	0.13	105	0.05
	c	113	0.23	107	0.4	105	0.55	N/A	0.05	102	0.03	103	0.3	105	0.06
	d	N/A	N/A	N/A	N/A	N/A	N/A	N/A	N/A	N/A	N/A	N/A	N/A	N/A	N/A
4.5	a	117	0.38	115	0.43	113	1.52	107	0.22	105	0.09	109	0.31	107	0.13
	b	N/A	N/A	N/A	N/A	N/A	N/A	N/A	N/A	N/A	N/A	N/A	N/A	N/A	N/A
	c	113	0.26	106	0.44	104	0.46	104	0.04	102	0.03	103	0.24	105	0.05
	d	N/A	N/A	N/A	N/A	N/A	N/A	N/A	N/A	N/A	N/A	N/A	N/A	N/A	N/A
5.5	a	110	0.29	109	0.6	107	1.19	104	0.06	102	0.03	104	0.22	105	0.06
	b	N/A	N/A	N/A	N/A	N/A	N/A	N/A	N/A	N/A	N/A	N/A	N/A	N/A	N/A
	c	108	0.32	103	0.19	103	0.15	104	0.04	102	0.03	102	0.11	105	0.05
	d	N/A	N/A	N/A	N/A	N/A	N/A	N/A	N/A	N/A	N/A	N/A	N/A	N/A	N/A
6.5	a	106	0.34	103	0.19	103	0.12	104	0.04	102	0.03	102	0.09	105	0.05
	b	113	0.3	112	0.49	112	0.57	111	0.06	110	0.03	110	0.14	113	0.07
	c	110	3.13	117	4.42	104	1.34	104	0.12	113	7.9	103	0.15	106	0.34
	d	N/A	N/A	N/A	N/A	N/A	N/A	N/A	N/A	N/A	N/A	N/A	N/A	N/A	N/A
7	Above stripper packing	108	0.12	110	0.12	109	0.21	110	0.09	109	0.04	109	0.08	112	0.09
8	Top stripper outlet	97	0.12	98	0.11	97	0.15	99	0.05	97	0.07	97	0.11	100	0.07

a There are four parallel temperature sensor, where the legends A, B, C, and D refer to the temperature sensor close to the column wall and inside the packing at horizontal 1 m distance from each other. N/A: Data not available.

**Table 5:** Stripper temperature profiles for LVC cases.

h (m)	Position of temperature probe^a^	Case 2A		Case 2B		Case 2C-1		Case 2C-2		Case 2C-3		Case 2D-1		Case 2D-2		Case 2E		Case 2F	
		T (°C)	Std. Dev. (°C)	T (°C)	Std. Dev. (°C)	T (°C)	Std. Dev. (°C)	T (°C)	Std. Dev. (°C)	T (°C)	Std. Dev. (°C)	T (°C)	Std. Dev. (°C)	T (°C)	Std. Dev. (°C)	T (°C)	Std. Dev. (°C)	T (°C)	Std. Dev. (°C)
0		121	0.01	119	0.04	117	0.05	117	0.11	118	0.02	115	0.05	115	0.05	116	0.05	118	0.03
0.5	a	120	0.05	116	0.1	112	0.43	113	0.41	114	0.09	102	0.24	103	0.26	113	0.11	110	0.13
	b	120	0.02	118	0.05	115	0.14	115	0.21	116	0.07	110	0.27	111	0.35	114	0.07	114	0.11
	c	119	0.02	115	0.14	109	0.27	111	0.45	112	0.19	98	0.2	100	0.25	111	0.13	111	0.18
	d	N/A	N/A	N/A	N/A	N/A	N/A	N/A	N/A	N/A	N/A	N/A	N/A	N/A	N/A	N/A	N/A	N/A	N/A
1.5	a	118	0.03	112	0.2	103	0.41	105	0.83	107	0.27	94	0.08	97	0.1	107	0.31	107	0.1
	b	119	0.02	117	0.09	111	0.26	112	0.45	113	0.1	100	0.21	102	0.34	112	0.09	110	0.17
	c	118	0.03	109	0.34	99	0.26	103	0.69	103	0.32	93	0.06	95	0.08	105	0.31	107	0.16
	d	117	0.04	105	0.34	97	0.18	100	0.56	99	0.16	93	0.05	96	0.05	101	0.3	106	0.11
2.5	a	118	0.04	113	0.19	103	0.45	106	0.89	107	0.27	94	0.12	97	0.12	108	0.27	107	0.17
	b	118	0.02	108	0.34	97	0.19	100	0.73	99	0.2	93	0.05	95	0.05	102	0.35	106	0.09
	c	N/A	N/A	N/A	N/A	N/A	N/A	N/A	N/A	N/A	N/A	N/A	N/A	N/A	N/A	N/A	N/A	N/A	N/A
	d	119	0.06	101	0.36	98	0.11	100	0.3	99	0.11	96	0.04	98	0.05	100	0.25	108	0.07
3.5	a	116	0.07	100	0.55	96	0.1	98	0.28	96	0.08	93	0.04	95	0.05	97	0.2	105	0.07
	b	115	0.08	95	0.13	95	0.09	97	0.15	95	0.04	93	0.05	95	0.05	95	0.09	105	0.05
	c	111	0.24	95	0.11	95	0.09	97	0.15	95	0.03	93	0.05	95	0.05	95	0.09	105	0.06
	d	N/A	N/A	N/A	N/A	N/A	N/A	N/A	N/A	N/A	N/A	N/A	N/A	N/A	N/A	N/A	N/A	N/A	N/A
4.5	a	115	0.16	97	0.35	98	0.09	99	0.18	97	0.04	95	0.03	97	0.07	97	0.09	107	0.13
	b	N/A	N/A	N/A	N/A	N/A	N/A	N/A	N/A	N/A	N/A	N/A	N/A	N/A	N/A	N/A	N/A	N/A	N/A
	c	110	0.35	95	0.09	95	0.08	97	0.14	95	0.03	93	0.04	95	0.05	95	0.08	105	0.05
	d	N/A	N/A	N/A	N/A	N/A	N/A	N/A	N/A	N/A	N/A	N/A	N/A	N/A	N/A	N/A	N/A	N/A	N/A
5.5	a	109	0.32	94	0.08	95	0.09	97	0.13	95	0.03	93	0.05	95	0.05	95	0.07	105	0.06
	b	N/A	N/A	N/A	N/A	N/A	N/A	N/A	N/A	N/A	N/A	N/A	N/A	N/A	N/A	N/A	N/A	N/A	N/A
	c	101	0.39	94	0.06	95	0.08	97	0.13	95	0.03	93	0.05	95	0.05	95	0.07	105	0.05
	d	N/A	N/A	N/A	N/A	N/A	N/A	N/A	N/A	N/A	N/A	N/A	N/A	N/A	N/A	N/A	N/A	N/A	N/A
6.5	a	100	0.27	94	0.07	95	0.08	97	0.13	95	0.03	93	0.05	95	0.05	95	0.07	105	0.05
	b	104	0.26	102	0.11	103	0.09	104	0.13	102	0.03	100	0.05	103	0.06	102	0.07	113	0.07
	c	105	5.38	95	0.96	131	26.54	97	0.12	95	0.04	93	0.03	97	0.18	95	0.06	106	0.34
	d	N/A	N/A	N/A	N/A	N/A	N/A	N/A	N/A	N/A	N/A	N/A	N/A	N/A	N/A	N/A	N/A	N/A	N/A
7	Above stripper packing	99	0.09	99	0.08	100	0.07	102	0.1	99	0.04	98	0.08	100	0.13	100	0.06	112	0.09
8	Top stripper outlet	89	0.09	89	0.05	89	0.08	91	0.11	89	0.04	87	0.08	89	0.16	89	0.06	100	0.07

a There are four parallel temperature sensor, where the legends A, B, C, and D refer to the temperature sensor close to the column wall and inside the packing at horizontal 1 m distance from each other. N/A: Data not available.

## 2 Experimental design, materials, and methods

A lean vapor compressor (LVC) campaign was performed at Technology Centre Mongstad using 30 wt% aqueous monoethanolamine (MEA) and flue gas, with a CO_2_ content of 3.5% supplied by the combined heat and power (CHP) plant at the nearby Equinor refinery.

The amine plant was designed and constructed by Aker Solutions and Kværner. The LVC compressor (Pinnacle LF2140 single stage) was manufactured by Sundyne Compressors. The packing height of absorber and stripper were 18 m and 8 m respectively. Both columns were packed with structured Flexipac 2X.

**Fig. 1:**
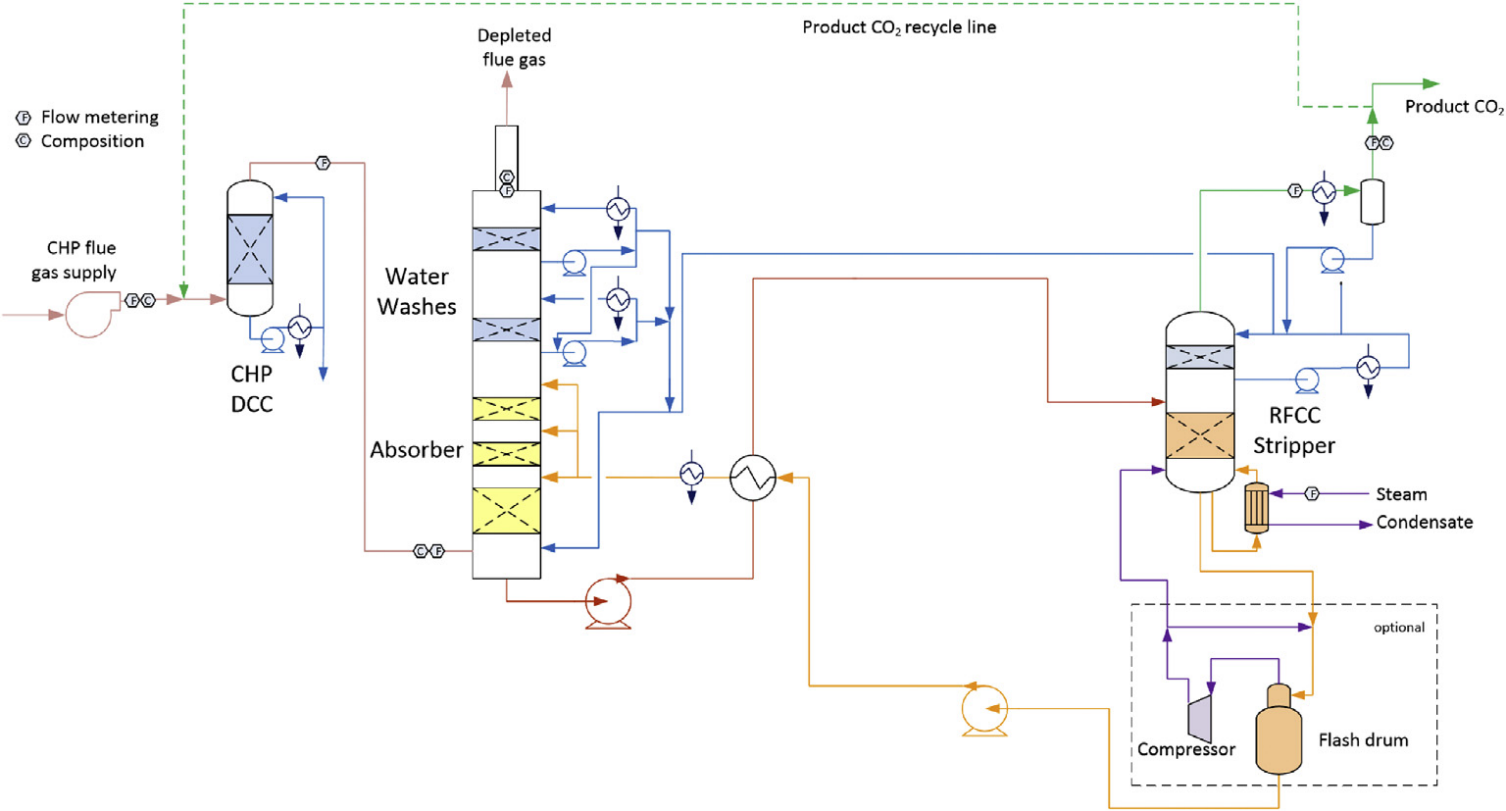
Simplified process flow diagram of the TCM amine plant.

A simplified process flow diagram illustrating the TCM amine plant configuration with CCGT based CHP flue gas feed, CO_2_ recycle, and the large stripper designed for high CO_2_ content flue gas is exemplified in Fig. 1. This set-up was utilized in the LVC test campaign.

The CHP flue gas is conditioned in a direct contact cooler (DCC) after being enriched with CO_2_ from the CO_2_ product recycle stream. The conditioned flue gas is contacted counter-currently with amine solvent in the absorber. CO_2_ is absorbed, yielding a solvent rich in CO_2_ and a depleted flue gas with low CO_2_ content. The depleted flue gas is released to the atmosphere after being conditioned in the water wash sections. The rich solvent loaded with CO_2_ is pre-heated in the lean/rich cross heat exchanger before entering the stripper column. Additional heat is supplied by steam to the stripper reboiler in order to desorb CO_2_ and regenerate the solvent. The product CO_2_ gas is released to the atmosphere, while the regenerated lean solvent is pumped back to the absorber via the lean/rich cross heat exchanger and the lean cooler. The amine plant is described in detail elsewhere [2], [3], [4].

**Fig. 2:**
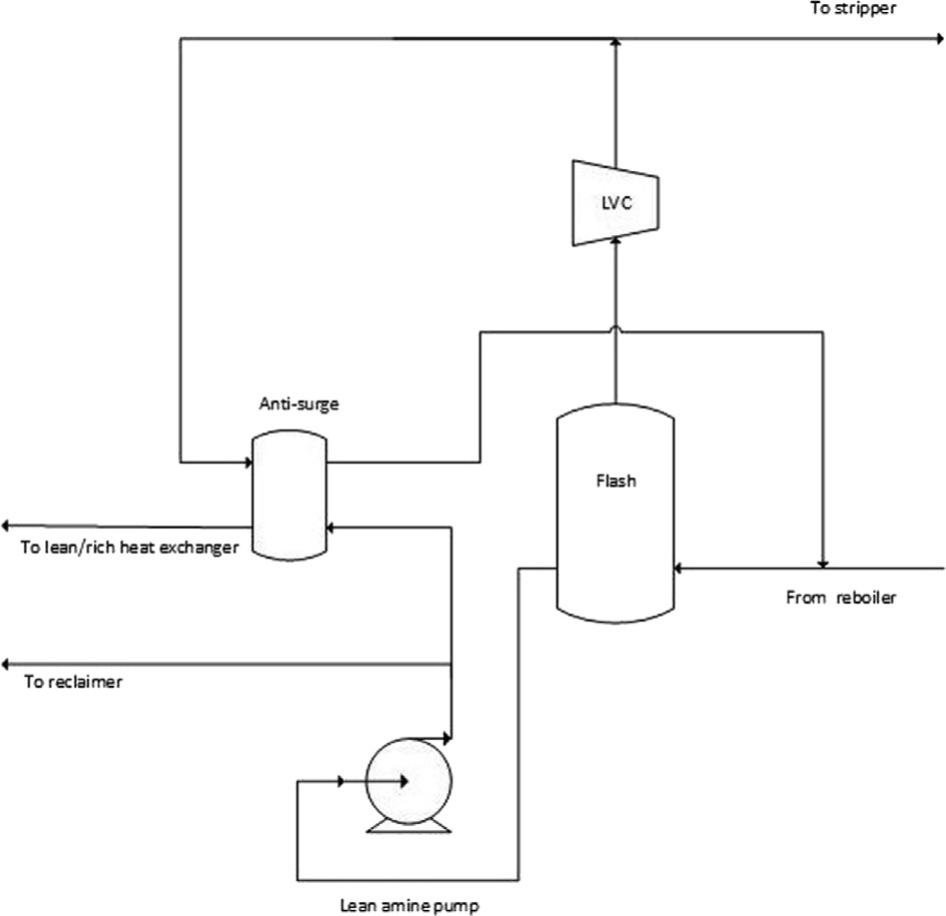
Simplified process flow diagram of the LVC.

The large stripper section designed for high CO_2_ content flue gas is also equipped with an optional lean vapor compressor system, as illustrated in Fig. 1. In the LVC system (see Fig. 2), hot lean amine exiting the stripper bottom is throttled to a lower pressure and fed to a flash drum generating vapor. The vapor is compressed and returned to the stripper bottom, while the lean amine is circulated back to the lean amine solvent loop. The LVC has for safety reasons a built-in anti-surge option which is used when flow to the compressor is below design flow. The control of the LVC automatically recycles gas in order to maintain correct compressor operation.

The superheated steam provides additional energy for regeneration of solvent in the stripper, which has the potential of reducing consumption of low pressure steam in the stripper reboiler.

Table 6 gives an overview of the adjustable process parameters applied in the LVC campaign at TCM.

**Table 6:** Overview of the LVC campaign with respect to basic process parameters used.

Case	Focus
1A to 1C	Solvent flow rate
1D	CO_2_ capture rate
1E	Stripper pressure
1F	Inlet flue gas CO_2_ concentration
2A to 2C-1	Solvent flow rate with LVC
2C-1 to 2D-2	CO_2_ capture rate and LVC pressure
2E	Stripper pressure with LVC
2F	Inlet flue gas CO_2_ concentration with LVC

The LVC campaign was operated in a way that only one parameter was adjusted at a time allowing the plant to reach steady state faster. The campaign was performed with case durations between 3 and 24 hours out of which 1–8 hours were used for calculation of average steady state conditions.
